# Theory-driven development of a medication adherence intervention delivered by eHealth and transplant team in allogeneic stem cell transplantation: the SMILe implementation science project

**DOI:** 10.1186/s12913-020-05636-1

**Published:** 2020-09-02

**Authors:** Janette Ribaut, Lynn Leppla, Alexandra Teynor, Sabine Valenta, Fabienne Dobbels, Leah L. Zullig, Sabina De Geest, the SMILe study team, Sonja Beckmann, Juliane Mielke, Anja Schmid, Nathalie Duerinckx, Phillip Heidegger, Margarita Fürmann, Daniela Neupert, Dennis Rockstein, Viktor Werlitz, Michael Fürmann, Tobias Schulz, Marina Lemcke, Vanessa Schumacher, Robert Zeiser, Monika Engelhardt, Monika Hasemann, Klaus Kaier, Sabine Gerull, Jakob Passweg, Anja Ulrich, Florian Grossmann, Dora Bolliger, Sigrun Reitwiessner, Sabine Degen, Sandra Schönfeld, Yuliya Senft, Birgit Maier, Chris Shultis

**Affiliations:** 1 Department Public Health, Faculty of Medicine, Institute of Nursing Science, University of Basel, Bernoullistrasse 28, 4056 Basel, Switzerland; 2 Department of Hematology, University Hospital of Basel, 4031 Basel, Switzerland; 3 Departments of Hematology, Oncology and Stem Cell Transplantation, University Medical Center Freiburg, 79110 Freiburg im Breisgau, Germany; 4 Department of Computer Science, University of Applied Sciences, Augsburg, Germany; 5 Academic Center for Nursing and Midwifery, Department of Public Health and Primary Care, University of Leuven, 3000 Leuven, Belgium; 6 Department of Population Health Science, Duke University, Durham, NC USA; 7 Center of Innovation to Accelerate Discovery and Practice Transformation (ADAPT), Durham Veterans Affairs Health Care System, Durham, NC USA

**Keywords:** Allogeneic hematopoietic stem cell transplantation, Medication adherence, Intervention development, Behavior change wheel, Theory-driven, eHealth intervention, Implementation science

## Abstract

**Background:**

Medication adherence to immunosuppressants in allogeneic stem cell transplantation (alloSCT) is essential to achieve favorable clinical outcomes (e.g. control of Graft-versus-Host Disease). Over 600 apps supporting medication adherence exist, yet they lack successful implementation and sustainable use likely because of lack of end-user involvement and theoretical underpinnings in their development and insufficient attention to implementation methods to support their use in real-life settings. Medication adherence has three phases: *initiation*, *implementation* and *persistence*. We report the theory-driven development of an intervention module to support medication adherence (implementation and persistence phase) in alloSCT outpatients as a first step for future digitization and implementation in clinical setting within the SMILe project (*Development, implementation and testing of an integrated care model in allogeneic* *SteM* *cell transplantatIon faciLitated by* *eHealth*).

**Methods:**

We applied Michie’s Behavior Change Wheel (BCW) and the Capability-Opportunity-Motivation and Behavior (COM-B) model using three suggested stages followed by one stage added by our team regarding preparation for digitization of the intervention: (I) Defining the problem in behavioral terms; (II) Identifying intervention options; (III) Identifying content and implementation options; (IV) SMILe Care Model Prototype Development. Scientific evidence, data from a contextual analysis and patients’/caregivers’ and clinical experts’ inputs were compiled to work through these steps.

**Results:**

(I) *Correct immunosuppressant taking* and *timing* were defined as target behaviors. The intervention’s focus was determined within the COM-B dimensions Capability (*lack of knowledge, lack of routine*), Opportunity (*lack of cues, interruptions in daily routine*) and Motivation (*lack of problem solving, trivialization*). (II) Five intervention functions were chosen, i.e. *education*, *training, modelling, persuasion* and *enablement*. (III) Twenty-four behavior change techniques were selected, e.g., *goal setting*, *action planning* and *problem solving*. (IV) Finally, seventeen user stories were developed to guide the *SMILeApp’s software development process*.

**Conclusion:**

Our example on the theory-driven development of an intervention module in alloSCT delivered by eHealth and transplant team using a rigorous 3 + 1-stage approach based on BCW, COM-B and agile software development techniques, can be used as methodological guidance for other eHealth intervention developers. Our approach has the potential to enhance successful implementation and sustained use of eHealth solutions in real-life settings.

## Background

Allogeneic stem cell transplant (alloSCT) is a well-established, potentially curative treatment modality for various malignant and non-malignant hematological diseases in which a patient’s diseased blood-producing system is replaced by that of a healthy person [1]. These patients’ complex care needs demand innovation in care delivery [2]. As information technology widely available through smartphones has the potential to support patients in adopting and developing essential behaviors such as those surrounding medication adherence [2, 3], the National Institutes of Health Hematopoietic Cell Transplantation Late Effects Initiative [2] recommend that related interventions include the support of eHealth solutions.

**Fig. 1:**
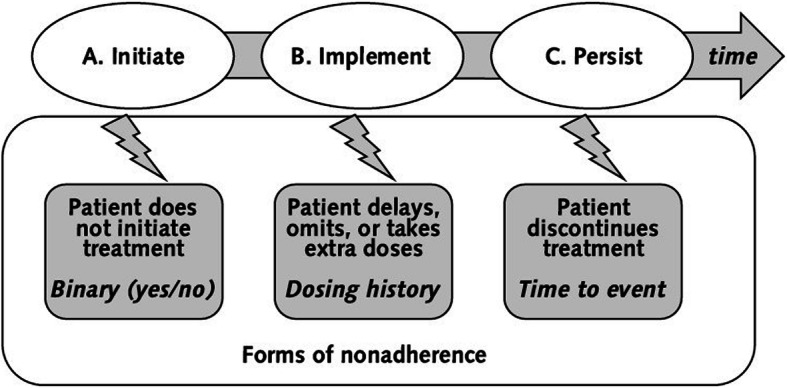
Concept of medication adherence with initiation, implementation and persistence [4], based on [5]. From Annals of Internal Medicine, De Geest S, Zullig LL, Dunbar-Jacob J, Helmy R, Hughes DA, Wilson IB, Vrijens B, ESPACOMP Medication Adherence Reporting Guideline (EMERGE), 169, 1, 30–35. Copyright© [2018] American College of Physicians. All Rights Reserved. Reprinted with the permission of American College of Physicians, Inc

Medication adherence is defined as “The process by which patients take their medications as prescribed, composed of *initiation*, *implementation* and *discontinuation*” (Fig. 1) ( [5], p. 697). *Initiation* means that the patient starts taking a prescribed medication. *Implementation* means the correspondence of a patient’s actual dosing compared to the prescription. *Implementation*^1^ errors can take the form of *late*, *skipped*, *extra*, or *reduced* doses or ‘*drug holidays*’ (skipping several doses in a row). If the patient quits the prescribed medication for any reason(s), this is called *discontinuation*. The time span between the first and last dose is called *persistence* [5].

Medication adherence is vital regarding clinical outcomes in patients following alloSCT [6, 7]: as many as 80% of alloSCT patients develop an acute or chronic Graft-versus-Host Disease (GvHD) [8, 9], a serious complication in which the donor’s immune cells attack the recipient’s body [1] leading to increased morbidity and mortality [8–11]. Although there is limited evidence and the few existing studies did not distinguish between the three medication adherence phases (initiation, implementation, persistence), we found medication adherence to immunosuppressants in alloSCT essential to prevent or treat complications such as acute and chronic GvHD [12, 13]. The correlation is extremely compelling: In patients without chronic GvHD only 15.6% showed medication non-adherence to immunosuppressants while in patients with mild GvHD already 66.7% were non-adherent. In patients with moderate GvHD 74.1% showed medication non-adherence and in patients with severe GvHD even 88.9% were non-adherent [12]. As alloSCT receive their first doses of immunosuppressants during their hospitalization, the *initiation* phase of adherence is not an issue [6, 7]; adherence enhancing interventions need to focus on post-discharge *implementation* and *persistence* of medication intake [12]. While available evidence on medication adherence in alloSCT populations is limited, we know that the prevalence of overall non-adherence to immunosuppressants in adult alloSCT patients is 64.6% with 33.3% taking, 61.2% timing and 4.1% dosing non-adherence, 3.2% drug holidays and 3.1% discontinuation [12].

Findings from randomized controlled trials (RCT) in other chronic disease areas, including solid organ transplantation, positively link behavioral interventions using eHealth with improvements in medication adherence [14–16], unplanned inpatient acute care admissions [16], costs [16], general health outcomes (e.g., blood glucose or blood pressure) [17] and overall quality of life [16, 17]. The most successful behavior change techniques (BCTs) (i.e., active intervention elements) used in these trials were self-monitoring of and feedback on medication intake [15–19], reminder cues [15, 18, 19], goal setting, action planning and problem solving [14–17, 20]. To date, though, no interventions focusing on these elements have been developed and tested in alloSCT.

Moreover, compared to the wealth of findings emerging from trials, comparatively little information is available on successful implementation and subsequent evaluation of medication adherence interventions in general and eHealth supported behavioral interventions in particular in real-world settings. A 2018 review on high quality medication adherence intervention trials in different patient populations found that most of them did not report on essential implementation research elements, hindering the adaptation and implementation of trial findings into real-world setting [21]. Another 2018 review paper found 681 apps to support medication adherence in the Apple App and Google Play Stores [22]. However, only 84 (12.3%) of these were developed by or in collaboration with health care professionals, only eight (1.2%) were theory-based, and none reported patient involvement in their development process [22]. And regardless of whether they were developed based on a specific theory, roughly two-thirds contained zero to two BCTs. Of apps including BCTs, 96% included reminders – which are often unneeded or unwanted by patients [23]. Some higher-value BCTs such as self-monitoring or feedback on behavior were included in a minority (36–39%), but techniques such as social support or information about the health consequences were very rare (1–2%) [23]. Combined with developers’ general omission of end-user involvement (especially from patients) [22, 24], general shortages both of theoretical underpinnings [2, 25] and of contextual information to adapt eHealth interventions for implementation in specific settings [26–28] very likely contribute to two major concerns: 1) More than two-thirds of patients who use health-related apps abandon them within 3 months [24]; and 2) Very few health behavior support interventions are ever successfully implemented in real-world settings [15, 18, 19, 29–32].

In order to support post-alloSCT medication adherence effectively and sustainably, eHealth supported medication adherence interventions need to be planned from start to finish with a clear focus on one overarching outcome: implementation in daily clinical alloSCT practice. Accordingly, our major goal with this paper is to report the development of an eHealth facilitated care medication adherence module (implementation and persistence phase) for alloSCT. To do so, we have employed the Behavioral Change Wheel (BCW) and the Capability-Opportunity-Motivation and Behavior (COM-B) model – both of which, unlike other behavioral theories (e.g. Theory of Planned Behavior and Health Belief Model [33]), provide multilevel perspectives and include emotional factors [34]. In addition, we have developed user stories that facilitate the translation of research findings first to digitalization of the intervention, then to standard practice among both clinicians and patients.

## Methods/results

### The SMILe project

This work is part of the international, interdisciplinary, multi-phase, multicenter SMILe project, which aims at reengineering follow-up care to patients with alloSCT into an integrated care model first at the University Medical Center Freiburg, Germany and in a second step at the University Hospital of Basel, Switzerland (https://nursing.unibas.ch/de/forschungsprojekte/forschung/forschung/smile/). The SMILe integrated care model builds on the eHealth Enhanced Chronic Care Model (eCCM) [3]. Its major aim is to introduce follow-up based on a chronic care model by providing self-management support, optimizing delivery system design (e.g., increasing continuity of care), clinical decision support and clinical information systems.

As these functions involve considerable time and expertise, we recommend the introduction of a Care-coordinator (CC) to perform them, along with the use of a SMILe e-platform including the SMILeApp and the SMILeCare monitoring component for the CC. For the first year post-alloSCT, in addition to connecting patients to the CC in the transplant center, the app will allow patients to assess relevant biomedical and behavioral parameters on a continuous basis.

Four intervention modules are integrated to support self-management and promote alloSCT patients’ target health behaviors: *Monitoring and Follow-Up*, *Infection Prevention*, *Physical Activity* and *Medication Adherence*. This paper reports on the development of the medication adherence module. This patient-centered, theory-based development approach will increase the likelihood of adoption and implementation in practice.

### Guiding methodology

**Fig. 2:**
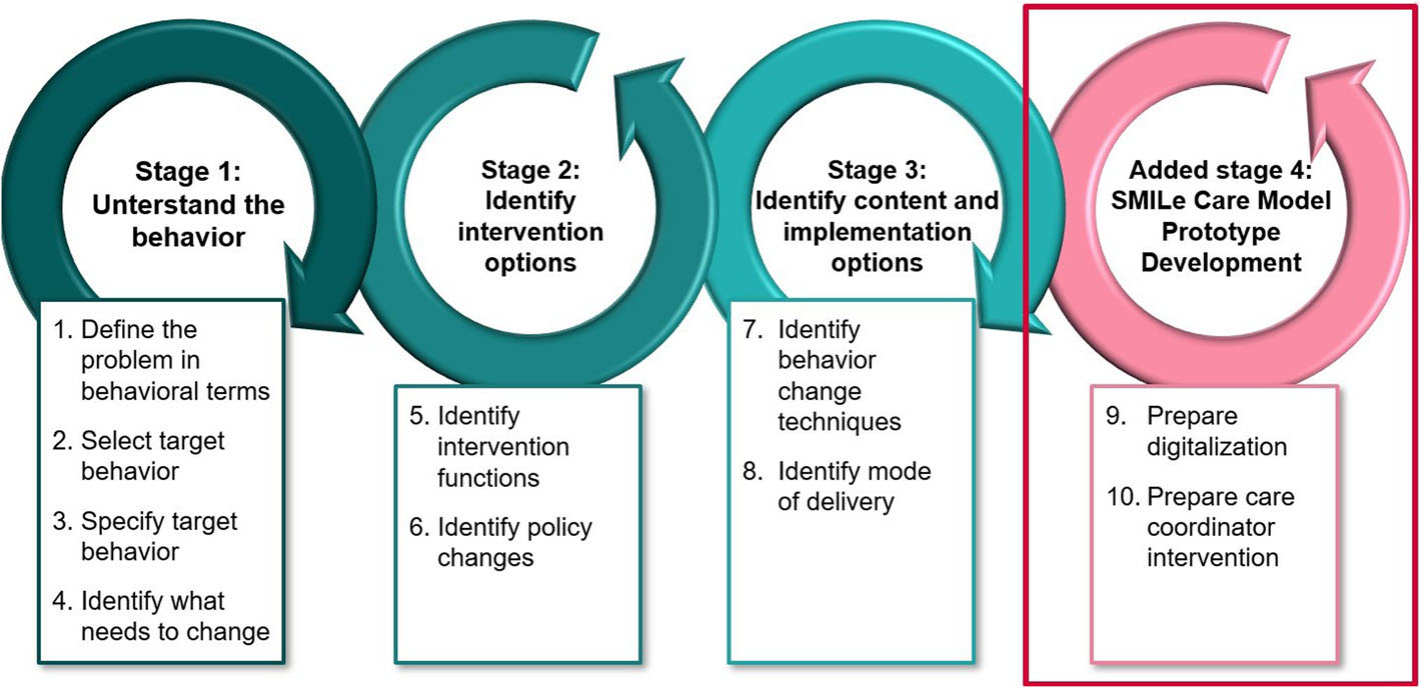
The 3 stages and 8 steps of the BCW [34] with our added stage 4 (self-developed figure)

Our intervention development process used an iterative approach applying the three stages of the BCW and one additional stage for the SMILe Care Model Prototype Development (Fig. 2), with each stage of execution reviewed by the entire SMILe research team [34, 35]. The various stages and steps will be further described in the next section (“The BCW”). Intervention development was informed by data from our previously performed multi-methods contextual analysis and evidence. Contextual analysis refers to the mapping of relevant multilevel contextual factors for the implementation of an intervention, e.g. local infrastructure, leadership, motivation of the stakeholders [27]. This means the inquiry of a specific context (in our case alloSCT follow-up care at the University Hospital of Freiburg, Germany), practice patterns as well as the attitudes and behaviors of all parties involved. Our preliminary work included such a contextual analysis using a cross-sectional quantitative survey of 60 alloSCT patients and five clinicians working in alloSCT. That analysis also included qualitative data, namely individual interviews with another ten alloSCT patients and three alloSCT clinician focus groups. The detailed description of the methods and results of this contextual analysis were recently published elsewhere [36]. It studied all relevant contextual aspects of the proposed integrated model of care, e.g., socio-cultural aspects at the micro (i.e., patient) and meso (i.e., transplant center) levels. Further, we examined the feedback of 21 stem cell transplant patients and eleven caregivers collected during three feedback rounds in Freiburg im Breisgau (Germany). Both, allogeneic and autologous stem cell transplant recipients were invited to give feedback as they participated a shared self-help group. We presented them our preliminary results and asked them to rank e.g. the importance of barriers to medication adherence or whether the proposed BCTs were feasible for them. The patients’ and caregivers’ feedback was included in the further development of the intervention. In addition, we reviewed existing evidence on 1) definition, prevalence and consequences of medication non-adherence in alloSCT (quantitative, appendix search diagram 1), 2) barriers and facilitators of medication adherence in alloSCT and solid organ transplantation (qualitative, appendix search diagram 2), and 3) existing interventions to support medication adherence in alloSCT and solid organ transplantation (quantitative, appendix search diagram 3). Because evidence in alloSCT is very limited, but medication adherence tasks are similar in alloSCT and solid organ transplant patients, we also included pertinent literature from solid organ transplantation.

### The BCW

One particularly rigorous and widely useful system of developing and implementing behavior change interventions is the BCW [37, 38]. Combining 19 behavior change frameworks, the BCW is used to identify, understand and explain behaviors and their influencing factors. It consists of three stages: 1) *understanding the behavior*, 2) *identifying intervention options* and 3) *identifying content and implementation options*, which are further divided into eight steps (Fig. 2) [34].

### BCW stage 1: understand the behavior

#### Step 1: define the problem in behavioral terms

Before attempting to modify a behavior it is necessary to understand it in detail and to identify possible starting points for change [34]. Therefore, the first step of the BCW is to define the problem in behavioral terms using three leading questions: *What is the behavior?; Where does it occur?;* and *Who is involved?*

In order to answer these questions, we first deepened our understanding of the problem by performing a scoping review of studies reporting definitions, prevalences and consequences of medication non-adherence in alloSCT (appendix, search diagram 1). From the twelve studies we identified, we extracted information on definition and relevance of medication non-adherence.

**Results.** Particularly regarding immunosuppressive regimes we found medication adherence essential in alloSCT to prevent or treat complications such as acute and chronic GvHD [12, 13]. However, almost 65% of participating patients struggled with medication intake, especially immunosuppressants taking and timing (*implementation* phase of medication adherence) [12] (Table 1).

**Table 1:** Definition of the problem in behavioral terms

Leading question	Possible answer
What is the problem/ behavior?	Medication non-adherence, which is associated with poor clinical and economic outcomes [20, 39], pervades all chronically ill patient populations [12, 13, 29, 40]. Post-alloSCT patients are chronically ill. With a mean of 12 medications [41], their medication regimen is complex. Most important medications in the 1st year post-alloSCT are immunosuppressants and anti-infectious medications. Co-medications (e.g., antibiotics, antivirals, fungicide) are essential to prevent and treat infections [42]. Using the ABC taxonomy, medication adherence consists of three interrelated phases: initiation, implementation and persistence [5], the latter two being relevant after alloSCT. Implementation adherence includes correct taking, timing, dosing, no drug holidays and correct food considerations (e. g., no grapefruit juice) [5, 12]. While available evidence on medication adherence in alloSCT populations is limited, we know that the prevalence of overall non-adherence to immunosuppressants in adult alloSCT patients is 64.6%: 33.3% taking non-adherence, 61.2% timing non-adherence, 4.1% dosing non-adherence, 3.2% drug holidays and 3.1% discontinuation [12]. Non-adherence to immunosuppressants is strongly associated with GvHD [12]. Less is known in view of co-medication. And while only 57% of the adult alloSCT patients report perfect medication adherence to all prescribed drugs after alloSCT [43], non-adherence can be quite selective, e.g., in one study 17% of subjects discontinued antifungal prophylaxis prematurely [44]. Therefore, it is crucial to optimize adherence, especially to immunosuppressants, in adults after alloSCT (implementation and persistence).
Where does it occur?	After alloSCT, non-adherence occurs at the patients’ homes and / or where they are at the scheduled time of medication intake. Our contextual analysis showed that alloSCT patients within the target setting understood the importance of following their medication regimen. According to clinicians, though, medication adherence was not systematically assessed at the target transplant center. If assessed, asking the patients for intake, monitoring blood-levels or checking for rejection signs were reported to be the most used practices. Clinicians also noted a need for a qualified person, e.g., an Advanced Practice Nurse or dedicated CC, to coordinate follow-up [36].
Who is involved? Who is our target group?	The entire health and health-related network surrounding adult patients after alloSCT (family, friends, health care professionals, ...) [36]. Community dwelling **adult** post-alloSCT patients

*alloSCT* Allogeneic stem cell transplantation, *GvHD* Graft-versus-Host Disease, *CC* Care-coordinator

The contextual analysis data of our preliminary work (see Guiding methodology section) [36] indicated that alloSCT patients were aware of the importance of following the medication regimen closely, especially when family, friends and caregivers were involved in medication management. According to clinicians, medication adherence was not systematically assessed at the studied transplant center. They also acknowledged a need for a person such as an Advanced Practice Nurse (APN) to coordinate follow-up, i.e., a CC [36]. The insights obtained from the literature review and contextual analysis enabled us to define the problem in behavioral terms (Table 1).

#### BCW step 2: select target behavior

The second step is to create a list of behavioral components known to influence the problem. From this list, behaviors are selected based on their expected impact (when improved) on target outcomes, the likelihood that they can be changed, the extent of expected spillover effects on other health behaviors and their measurability [34].

**Results.** In the literature search we identified 12 quantitative studies about prevalence and consequences of different behavioral components (appendix, search diagram 1). Based on the information from that literature review, we compiled a list of potential target behaviors that influence *implementation* and *persistence* adherence. For example, *taking, timing and dosing immunosuppressants correctly (three behaviors)*, *following food considerations concerning immunosuppressants* and not taking *drug holidays* (*implementation* dimension)*.* From this list, we selected *correct taking* and *timing of immunosuppressants* as the two most prevalent and important target behaviors. This selection was guided by a systematic rationale: changing these behaviors was expected to have the best overall combination of direct impact on clinical outcomes, spillover effects on other behaviors, e.g., *taking no drug holidays,* and ease of measurement (Table 2). We based our rating system (i.e., ++ very promising, + promising, ± not promising but worth considering, − unacceptable) on evidence from the literature and the research team’s clinical expertise.

**Table 2:** List of possible target behaviors

Possible target behavior	Impact of behavior change	Likelihood of change	Spillover effect	Measurement
Correct taking of immunosuppressants (i.e. persistently reducing number of missed immunosuppressants doses)	++ (↓ risk of GvHD [12])	++	++ (adherence to co-medication, no drug holidays)	++ [45]
Correct timing of immunosuppressants	++? (↓ risk of GvHD? [46, 47])	+	++ (adherence to co-medication, no drug holidays)	++ [45]
Correct dosing of immunosuppressants	++ (↓ risk of GvHD [12])	+	+ (adherence to co-medication, no drug holidays)	± [45]
Performing no drug holidays	++ (↓ risk of GvHD [12])	++	+ (adherence to co-medication)	++ [45]
Following food considerations	++ (↓ risk of GvHD [12])	+	+ (considerations for co-medication)	- [45]

*EM* Electronic monitoring; *GvHD* Graft-versus-Host Disease

(++ very promising) (+ promising) (± not promising but worth considering) (− unacceptable)

#### BCW step 3: specify the target behavior

As the third step is to examine the selected behaviors (in our case, two) both from the patient’s perspective and within the context of the surrounding system [34], we specified each in terms of *who*, *what*, *when*, *where*, *how*, *how often* and *with whom* they occurred. This choice of details was based on previously evaluated evidence from the literature [12, 13, 41], but mainly on our contextual analysis [36] and our research team’s clinical expertise.

**Results.** The resulting details (Table 3) allowed identification of target behaviors [34].

**Table 3:** Specification of target behaviors

Target behavior	Correct taking of immunosuppressants	Correct timing of immunosuppressants
Who	Adult alloSCT patients	Adult alloSCT patients
What	**Take** immunosuppressants 2x/day	Take immunosuppressants at prescribed **time**
When	a.m. & p.m. (at least until day 120–180, mean 16 months after alloSCT)	e.g., 9^00^ & 21^00^, max. Deviation 2 h (at least until day 120–180, mean 16 months after alloSCT)
Where	Patient location (home, work, vacation, ...)	Patient location (home, work, vacation, ...)
How	Swallowing pills with fluid (e.g., water, no grapefruit juice)	Swallowing pills with some fluids (e.g., water, no grapefruit juice)
How often	Every day	Every day
With whom	Alone (possibly with support of family/friends, nurse)	Alone (possibly with support of family/friends, nurse)

*alloSCT* Allogeneic stem cell transplantation

#### BCW step 4: identify what needs to change

**Fig. 3:**
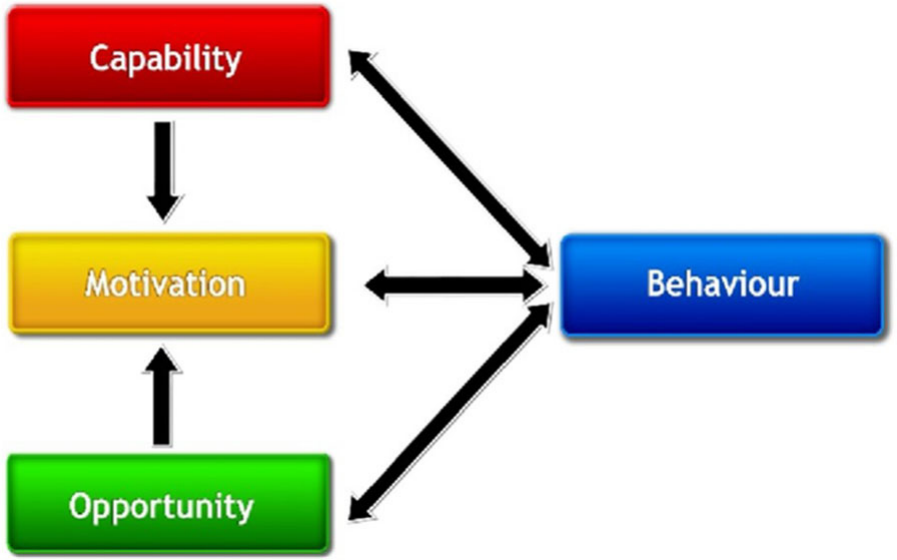
The COM-B framework to understand a behavior [34] (open access figure)

**Fig. 4:**
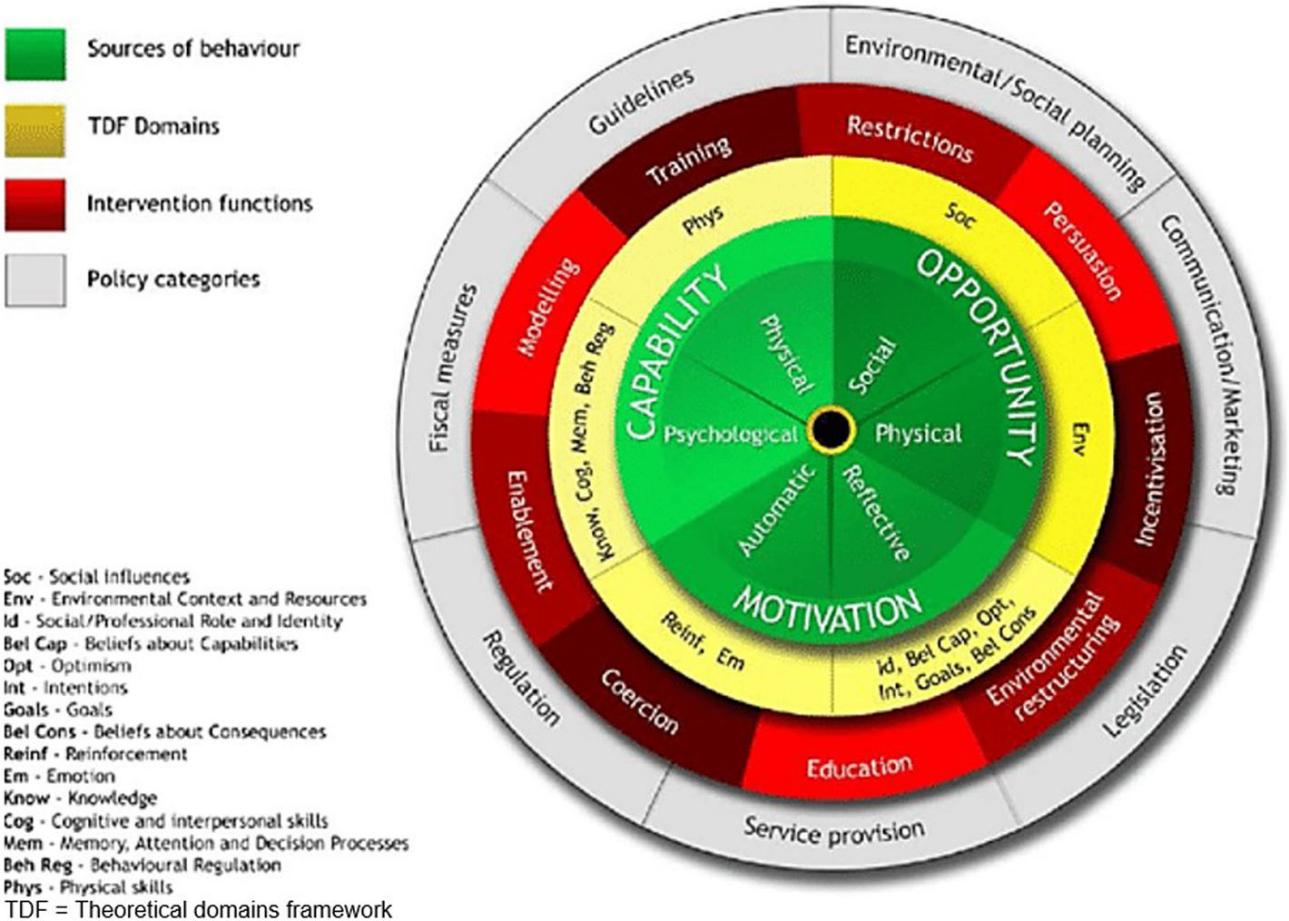
The behavior change wheel [34] (open access figure)

The fourth step is to identify what needs to change by analyzing risk factors not only at the individual but also higher in the system. For this step, the COM-B model (Fig. 3) – the center of the BCW (Fig. 4) – supports behavioral analysis very well, as it acknowledges that behaviors such as medication non-adherence are influenced by the physical and psychological *capability*, physical and social *opportunity* as well as reflective and automatic *motivation*. *Capability* is the capacity to engage in a certain health behavior (e.g., medication intake); *opportunity* refers to factors external to the individual that make behavior possible; and *Motivation* refers to the brain processes – whether reflective or unconscious – that direct behavior. Two common examples of *capability* are cognitive functionality (psychological capability) and the physical capability to swallow medication. *Opportunities* may lie in the accessibility of the medication (physical opportunity) or support from a partner (social opportunity); and *motivational* aspects would include attitudes about the medication, such as beliefs about its efficacy (reflective motivation) or treatment fatigue (automatic motivation).

Of course, capability and opportunity also affect motivation, and the behavior itself influences all three adherence components (Fig. 3). As an ecologic model (i.e., involving factors on the patient, health care provider, health care organization and health care system level), the BCW is helpful in understanding behaviors’ determinants and emotional drivers and provides the basis for the following steps.

In addition, the COM-B can be combined with the Theoretical Domains Framework (TDF) [34], which synthesizes multiple behavioral theories and includes 14 domains of behavioral influence (Fig. 4). Each domain represents theoretical constructs such as *knowledge*, *skills* or *beliefs* and relates to one COM-B component. Used together, the COM-B and the TDF allow a *behavioral diagnosis*, which facilitates the choice of effective behavior change interventions [34].

Where exactly to initiate behavior change was determined partly by the results of the contextual analysis and partly by qualitative evidence. We located the latter via a scoping review for qualitative findings on alloSCT patients’ and clinicians’ perspectives, barriers and facilitators to medication adherence. Due to limited evidence in the alloSCT population we also included literature from the solid organ transplant population (appendix, search diagram 2).

**Results.** According to the findings of the 16 identified qualitative studies and the contextual analysis, implementation of medication adherence is influenced by a broad set of multilevel factors, which we organized within the COM-B model (Table 4). For instance, *lack of knowledge* and *lack of routine* (Capability), *lack of cues* and *interruptions in daily routine* (Opportunity), *lack of problem solving*, *trivialization and denial* (Motivation) were identified as possible barriers to medication adherence implementation.

**Table 4:** Barriers of medication adherence sorted by COM-B

COM-B	TDF	What needs to happen for target behavior to occur?	Information about barriers to medication adherence	Do factors/barriers need to change to perform target behavior (based on contextual analysis)?
**Physical capability**	Physical skills	Being physically able to swallow pills and remember if already taken	Emesis, nausea [48, 49] ^a b^ Poor physical condition [50] ^a b^	NO Physical limitations (e.g. cognitive function) which can’t change
				YES Limited physical stamina (e.g. fatigue, nausea)
**Psychological capability**	Knowledge	Knowledge why intake and timing of immunosuppressants is important	Lack of knowledge about medication and consequences [48, 50–61] ^a b^ Wrong information ^b^ Ambiguities / vague advise [50, 52, 53] ^a^ Unclear, where getting prescription from and which pharmacy [49–51, 57, 62] ^a b^	YES Lack of knowledge about importance and consequences of medication (non-) adherence
	Behavioral regulation	Apply the knowledge of correct medication intake and timing in every aspect, applying *if-then-rules* Development of coping strategies for barriers	Lack of routine [51, 52, 55–57, 59, 63] ^a^ No sense of autonomy regarding medication intake [51] History of MNA [53, 64] Longer time since transplantation [64] Current major life event (other priorities) [49, 53] Busy lifestyle [48, 49, 51, 54, 57] Alcohol and substance abuse [51]	YES Lack of procedural knowledge of medication intake YES Lack of behavioral regulation (e.g. self-monitoring) YES Lack of skills to develop coping strategies facing barriers
	Memory, attention & decision processes	Notice and remember at prescribed time during daily life to take medication	Information overload ^b^ Forgetfulness [48, 49, 51, 52, 54–57, 65] ^a^, poor executive function after transplantation [53, 65] Forgetting get a new prescription on time [49, 50, 54, 56, 57, 60] ^a b^	YES Limitations in memory, concentration, attention & decision processes ⋄ Lack of awareness / recognition in daily life
	Cognitive and inter-personal skills	Development of habit in correct timing and intake of medication, Skill to ask for help if needed	Unable to cope with changed prescription [54] Lack of psychological skills to fill pill boxes / prepare medication correctly [60]	YES Lack of habit-forming, goal-setting or action-planning skills
**Physical opportunity**	Environ-mental context and resources	Enough medication available, readily accessible at opportunities, enough reminder clues	Not having medicines when being away from home [48, 49, 54, 57] ^a^ Lack of cues [48–50, 52, 55–57, 60] ^a b^ Time of intake «does not fit» to lifestyle [48–50, 56, 57, 65] ^a^ Interruptions in daily routine [48–50, 52, 54, 56, 57] ^a^ Distance to clinic, no regular follow up [50] Travelling (e. g. to other time zone) [48, 49, 57] Changed time of intake due to clinical visits implies that it is not important [56, 57] ^a^ Complexity due to polypharmacy [48, 50, 51, 53, 56, 61] ^a b^ Barriers to take immunosuppressants [62]	YES Lack of facilitation via accessibility, possibility, easiness, availability, convenience YES Lack of facilitation via support for memory, concentration, attention
**Social opportunity**	Social influence	Patient participation & empowerment regarding an increased awareness about that medication management is a shared duty of everyone involved in the care	Lack of family support (emotional, instrumental) [48–53, 55–57, 59, 61, 62, 65] ^a b^ Lack of social support [48, 49, 51, 53, 55, 56, 59, 61, 62, 65] ^b^ Lack of peer learning (blogs, internet forum, waiting room) [52, 53, 56, 61] ^a^ Incorrect lay knowledge from peers ^b^ Lack of positive and negative role models [58] Lack of individual support (by nurses, pharmacists) [50, 52, 53, 56] ^a^ Avoiding taking medication in public / in front of friends [56] Lack of comparison with worse ill people [56, 57] Lack of trusting partnership with health professionals [50, 53, 57, 59] Lack of attention from nurses / health care professionals [53, 55, 62]	YES Lack of positive role models YES Lack of social or peer support (e.g. patient empowerment / participation, private or, professional support) YES Lack of awareness that medication intake can be improved by of everyone involved in the care
**Automatic Motivation**	Emotion	Positive emotions related to medication adherence	Feeling overwhelmed [58, 63] ^b^ Burnout / treatment fatigue [48, 52, 55, 57]; Depression [51, 57, 65] Low quality of life (direction of association unclear) [51] Negative emotions / attitude [49, 53, 58, 59]; Distress [51, 54] Desire for independence in self-management [52, 65] Nuisance due to repetitive reminder [57] Low gratitude toward medical team/donor [48–52, 54–56, 61] Tablet phobia (fear of swallowing tablets) [53]^a^	YES Lack of coping strategies YES Low relationship with health care provider YES Fear of embarrassment
	Reinforcement	Strategies for possible problems	Incompatibility of the immunosuppressants ^a b^ Side effects [48–53, 65] ^a b^	YES Lack of problem solving strategies
**Reflective Motivation**	Intentions	Have willingness and a plan on correct intake and timing	Lack of intention to adhere [51] ^a^ Not interested in learning about medication before transplant [60]	YES Insufficient intention YES Insufficient goals
	Beliefs: consequences	Correct beliefs of resulting consequences of non-adherence	Beliefs in illness, medication and side effects [48, 50–61, 63] ^a b^ Lack of knowledge about consequences [48–55, 57, 60] ^a^ Consequences of MNA not clear (health belief) [51, 55] ^a b^ Blood test did not capture MNA [56] Establishing a personal leeway of time [49] Defining acceptable risks [52] Trivialization and denial [52, 56, 57] ^a^	YES False beliefs about consequences
	Beliefs: capabilities	Correct beliefs of capability in medication management	No confidence in self-management (mastery) [50–53, 58] Lack of problem solving competence and self-efficacy [50–52] ^a^	YES False beliefs about own capabilities (e.g. self-efficacy)
	Goals	Correct beliefs of own responsibility for outcomes	Lack of motivation to convalescence [55, 58] ^b^ Making medications a low priority [57]	YES False beliefs in own responsibility for wanted outcomes
	Optimism	Confidence that desired goals will be achieved	Maladaptive coping [65]	YES Lack of adaptive coping strategies (to reduce stress)
	Role and identity	Compatible set of behaviors with professional identity	Evading patient hood [52] Seeing self as a victim [52]	YES Behaviors incompatible with professional identity

*MNA* Medication non-adherence

^a^: 10 individual interviews with alloSCT patients conducted by our research team, 2017; ^b^: 3 focus groups with alloSCT health care providers conducted by our research team, 2017

We crosschecked and compared our review’s results with those of two recent quantitative studies on multilevel determinants of medication non-adherence in heart transplantation [62]. In addition, we analyzed data from a long-term bio-psychosocial follow-up in solid organ transplantation [64] in which our research group was involved. The results supported our previous findings.

According to the findings of the 16 identified qualitative studies [48–61, 63, 65], the results of our contextual analysis and patient feedback, we selected the highest-priority barriers. The *behavioral diagnosis* findings for each domain and necessary changes are listed in Table 4.

### BCW stage 2: identify intervention options

#### BCW step 5: identify intervention functions

After identifying the target needs, the relevant intervention functions (Fig. 4) must be identified. The BCW lists nine of these (*education*, *persuasion*, *incentivization*, *coercion*, *training*, *restriction*, *environmental restructuring*, *modeling*, *and enablement*), each of which is a set of effective intervention categories with the potential to mitigate barriers to behavior change identified by the COM-B and/or TDF models.

To guide our selection, we applied the APEASE criteria (test of Affordability, Practicability, Effectiveness and cost-effectiveness, Acceptability, Side effects/safety, and Equity) [66] on each intervention function (Table 5). The identified components were rated (i.e., ++ very promising, + promising, ± not promising but worth considering, − unacceptable) with consideration for available evidence, the research team’s clinical expertise, our contextual analysis and patient feedback. The APEASE criteria were also applied in further steps, always using the same data sources mentioned here. To search for existing interventions that support medication adherence in alloSCT, we performed a systematic literature search (appendix, search diagram 3). Due to limited evidence in the alloSCT setting, we also included findings from the solid organ transplant population.

**Table 5:** Applying the APEASE criteria to select useful intervention functions

Factors/barriers that need to change to perform target behavior (step 4)	Intervention function	Affordability	Practicability	(Cost-) Effectiveness	Acceptability	Side-effects/ Safety	Equity	Does the intervention function meet the APEASE criteria, comments why yes/no?
**PhC:** Lack of knowledge; lack of awareness / recognition in daily life **ReM:** False beliefs about consequences and own responsibility for intended outcomes	Education	++	++	+	++	+	+	YES – provision of knowledge
**SoO:** Lack of awareness that medication intake can be improved by everyone involved in care **AuM:** Poor relationship with health care provider; fear of embarrassment **ReM:** False beliefs about own capabilities; behaviors incompatible with identity	Persuasion	+	+	+	++	++	++	YES – foster positive feelings, motivation; beliefs about capability, goals (self-efficacy)
	Incentivization	–	±	–	±	+	±	NO – not effective and not affordable
	Coercion							NO – Not acceptable
**PsC:** Lack of knowledge of drug intake procedure; lack of skills to develop coping strategies; limitations in memory & decision processes **PhO:** Lack of facilitation via support for memory, concentration, attention **SoO:** Lack of social or peer support **AuM:** Lack of coping strategies **ReM:** Lack of adaptive coping strategies	Training	+	+	+	+	++	++	YES – provision of training to prepare medication / use pill boxes / use reminders / self-monitoring
	Restriction							Not applicable
	Environmental restructuring	±	±	±	±	+	+	NO – not affordable (provision of administration aids)
**SoO:** Lack of positive role models	Modelling	+	+	+	++	++	++	YES – provision of peer learning, video
**PhC:** Limited physical stamina **PsC:** Lack of habit-forming, goal-setting or action-planning; lack of behavioral regulation **PhO:** Lack of facilitation via accessibility, easiness, availability, convenience **SoO:** Lack of social or peer support **AuM:** Lack of problem-solving strategies **ReM:** Insufficient intention and goals	Enablement	+	+	+	+	++	++	YES – skills and strategies to deal with barriers (e.g., side effects, interruptions in daily routine)

*PhC* Physical Capability, *PsC* Psychological Capability, *PhO* Physical Opportunity, *SoO* Social Opportunity, *AuM* Automatic Motivation, *ReM* Reflective Motivation

(++ very promising) (+ promising) (± not promising but worth considering) (− unacceptable)

**Results.** According to eleven quantitative studies we identified on medication adherence-enhancing interventions, combinations of *education*, *training*, *enablement*, *modeling*, *environmental restructuring* and *persuasion* were effective functions to support medication adherence [15, 18–20]. After considering the feasibility of each according to our contextual analysis, we excluded *environmental restructuring,* as this was not feasible within the SMILe project’s context. We then presented the selected functions to alloSCT patients and caregivers to discuss their feasibility and usefulness. The research team applied the APEASE criteria to the functions favored by the patient and caregiver group. They found that *education*, *training*, *enablement*, *modeling* and *persuasion* to have the greatest potential to improve medication adherence in post-alloSCT patients (Table 5).

#### BCW step 6: identify policy categories

In this step, policy categories are identified (Fig. 4). The BCW lists seven such categories (*communication/marketing*, *guidelines*, *fiscal measures*, *regulation*, *legislation*, *environmental/social planning*, *service provision*), from which researchers can choose those that will best support delivery of the intervention functions selected in step 5 [34]. We based our selection of policy categories on the results of our contextual analysis.

**Results.** After applying the APEASE criteria once again we selected two policy categories – *regulation* and *service provision* – as those most congruent with our chosen intervention functions (Table 6).

**Table 6:** Applying the APEASE criteria to select useful policy categories based on contextual analysis

Intervention function	Policy category	Affordability	Practicability	(Cost-)Effectiveness	Acceptability	(Side-effects) / Safety	Equity	Does the intervention function meet the APEASE criteria, comments why yes/no?
	Communication/ marketing	±	+	±	++	+	+	NO – not affordable, not suitable in our situation
	Guidelines	–	±	±	+	++	++	NO – national level ➔ not affordable in our situation
	Fiscal measures	–	±	±	±	+	++	NO – not affordable in our situation: Germany: Financial burden due to travelling to Tx center, drug *usually* paid Switzerland: Patients pay *Deductible* + 10%
Education, Persuasion, Training, Modeling, Enablement	Regulation	+	+	+	+	+	++	YES – center level ➔ regulation about what, how & how long contact with health care provider can continue
	Legislation							NO – not relevant, not practicable in our setting
	Environmental/ social planning	±	±	+	+	+	++	NO – WLAN access ➔ not affordable in our situation
Education, Persuasion, Training, Modelling, Enablement	Service provision	++	++	+	++	++	++	YES – e.g., support & checks via phone / improvement of performance at visits, introduction of a CC

*CC* Care-coordinator

(++ very promising) (+ promising) (± not promising but worth considering) (− unacceptable)

### BCW stage 3: identify content and implementation options

#### BCW step 7: identify behavioral change techniques

The seventh step is to identify BCTs, which are the active elements of an intervention. The BCT Taxonomy describes 93 BCTs, classified in 19 categories (e.g., *goal setting*, *action planning*, *problem solving*, *information about health consequences*, *self−/monitoring of behavior*, *self-reward*, *punishment*) to change a behavior. Thanks to the BCT Taxonomy, uniform terminology can also be used to describe intervention components, allowing replication of a study [34]. Based on the findings of the previous BCW steps and the data sources explained above, we selected the BCTs that we judged would best support our chosen intervention functions.

**Results.** In the literature search, we identified 11 quantitative studies on interventions (appendix, search diagram 3). According to the results, cognitive-educational interventions (e.g., information and instructions) were frequent, but showed inconsistent results in enhancing medication adherence [18, 20]. Behavioral interventions (e.g., counseling, reminders, self-monitoring and feedback on medication intake) led to significant improvements in medication adherence [15, 18–20]. While combinations of cognitive-educational, behavioral and psychological-affective interventions showed the best improvements, even these results were inconsistent [18, 19]. To select suitable BCTs for our intervention, we applied the APEASE criteria to those we judged most promising (Table 7).

**Table 7:** Applying the APEASE criteria to select useful BCTs in relation to COM-B and TDF

COM-B		TDF	Intervention function	Potential BCTs	Affordability	Practicability	(Cost-) Effectiveness	Acceptability	(Side-effects) / Safety	Equity	Does the BCT meet the APEASE criteria?
Capability	Physical	Physical skills	Enablement	1.4. Action planning (what to do when nausea)	+	+	++	+	++	++	yes
				11.1. Pharmacological support	±	+	++	++	+	++	yes
	Psychological	Knowledge; Behavioral regulation; Memory, attention and decision processes; Cognitive and interpersonal skills	Education Training Enablement	5.1. Information about health consequences	++	++	++	++	++	++	yes
				2.7. Feedback on outcomes of the behavior	+	+	+	++	++	++	yes
				8.3. Habit formation (at real opportunities)	+	+	+	+	++	++	yes
				8.4. Habit reversal (at real opportunities)	±	+	±	±	±	±	no
				1.1. Goal setting	+	+	+	+	++	++	yes
				1.4. Action planning	+	+	+	++	++	++	yes
				1.6. Discrepancy between current behavior & goal	+	+	+	±	+	++	yes
				1.2. Problem solving including coping planning	+	+	+	++	++	++	yes
Opportunity	Physical	Context and resources	Enablement Training	12.5. Adding objects to the environment	+	+	++	+	++	++	yes
				7.1. Prompts/cues	++	++	++	+	+	++	yes
				12.1. Restructuring of the physical environment	–	–	±	–	±	+	no
				4.1. Instruction on how to perform behavior	++	++	++	++	++	++	yes
	Social	Social influence	Training Persuasion Enablement	2.2. Feedback on behavior	++	++	++	++	++	++	yes
				2.3. Self-monitoring of behavior	++	++	++	+	++	++	yes
				6.1. Demonstration of behavior	+	+	+	+	++	+	yes
				3.1.-3.3. Emotional, practical & unspecified social support	+	+	++	+	+	+	yes
Motivation	Automatic	Emotion	Persuasion	15.3. Focus on past success	+	+	++	++	++	++	yes
			Enablement	11.2. Reduce negative emotions	±	±	±	++	++	++	no
		Reinforcement	Training	8.1. Behavioral practice / rehearsal	++	+	++	++	++	++	yes
				15.4. Self-talk	++	++	++	+	++	++	yes
	Reflective	Intentions; Beliefs about consequences / capabilities; Goals; Optimism; Social role & identity	Persuasion Enablement Training	9.1. Credible source	+	+	+	+	++	++	yes
				16.2. Imaginary reward	++	+	++	+	++	++	yes
				9.2. Pros & Cons	+	+	++	+	++	+	yes
				15.1. Verbal persuasion about capability	+	+	+	+	++	++	yes

(++ very promising) (+ promising) (± not promising but worth considering) (− unacceptable)

Based on these findings, we prepared a draft of the intervention, i.e. visualized the selected BCTs (Table 7) including a short description and a self-drawn representation of an app on a PowerPoint presentation. We showed the presentation to stem cell transplant patients and caregivers and discussed the various parts’ feasibility and usefulness. The participants had the possibility to vote for the different BCTs via a real-time smartphone-based survey (using mentmeter.com) as well as via the accompanying oral group discussion. The patients’ feedback indicated that they saw the intervention as both feasible and supportive. With consideration for all of the information thus far compiled, we selected 24 patient-level BCTs, including *goal setting*, *action planning* and *self-monitoring of behavior*. All selected BCTs are listed in Table 8.

**Table 8:** The selected intervention functions, policy categories, BCT and delivery mode relating to COM-B and TDF

COM-B		TDF	Intervention function	Policy category	BCT	Mode of delivery
Capability	Physical	Physical skills	Enablement	Service provision, Regulation	1.4. Action planning 11.1. Pharmacological support	Face-to-face, Phone helpline, info on app Mobile phone App (written information)/ Phone helpline with linkage to TX-Center
	Psychological	Knowledge Behavioral regulation Memory, attention & decision process Cognitive & interpersonal skill	Education Training Enablement	Service provision, Regulation	5.1. Information about health consequences 2.7. Feedback on outcomes of the behavior 8.3. Habit formation (at real opportunities) 1.1. Goal setting 1.6. Discrepancy between behavior & goal 1.2. Problem solving (incl. Coping planning)	Face-to-face, App (written, video) Face-to-face Face-to-face Face-to-face, App (goal-reminder) Face-to-face, Phone helpline Face-to-face, Phone helpline
Opportunity	Physical	Context & resources	Training Enablement	Service provision, Regulation	7.1. Prompts/cues 4.1. Instruction on how to perform behavior 12.5. Adding objects to the environment	App (reminder, information), Face-to-face Face-to-face, App (video, written info) App itself, information on App, Face-to-face
	Social	Social influences	Training Modelling Persuasion Enablement	Service provision, Regulation	2.2. Feedback on behavior 2.3. Self-monitoring of behavior 6.1. Demonstration of behavior 3.1.-3.3. Emotional, practical and unspecified social support	Face-to-face, App (by mobile phone text) App questionnaire, Face-to-face Face-to-face, App (video) Face-to-face
Motivation	Automatic	Emotion Reinforcement	Persuasion Enablement Training	Service provision, Regulation	15.3. Focus on past success 8.1. Behavioral practice / rehearsal 15.4. Self-talk	Face-to-face, Phone helpline Face-to-face Face-to-face, App (included in reminder)
	Reflective	Intentions Beliefs Goals Optimism Role & identity	Education Persuasion Enablement Training	Service provision, Regulation	9.1. Credible source 16.2. Imaginary reward 9.2. Pros & Cons 15.1. Verbal persuasion about capability	App (video) Face-to-face, Phone helpline Face-to-face Face-to-face, Phone helpline

#### BCW step 8: identify mode of delivery

The eighth and last of the BCW’s core processes is to identify the most suitable mode of delivery for the selected BCTs. This determines how the intervention should be delivered to the alloSCT patients and their caregivers, i.e., the end-users. A wide variety of delivery modes can support an intervention’s implementation and effectiveness, ranging *face-to-face individual* or *group sessions* to *mobile phone apps* [34]. The optimal delivery mode was determined using information generated from the previous steps, the contextual analysis and end-user feedback [34].

**Results.** Our contextual analysis [36] showed that alloSCT patients were open to technological assistance but emphasized that eHealth support should not replace personal contact with the health care team. Accordingly, of all available possibilities for face-to-face (delivered by the CC) or distance (technology assisted) interventions, we selected the most suitable mode of delivery for each of our intervention’s BCTs (Table 8).

The resulting draft of the SMILe Care Model Prototype integrates a CC (an APN with specialization in oncology) and the SMILeApp (“prototype” means the whole care model, not only the app component). The BCTs to support medication adherence will be delivered to the patients during two face-to-face visits with the CC, e.g. *demonstration how to perform the behavior* (e.g. prepare the medication) or *habit formation*. Depending on the patient’s condition, the care protocols allow for a step-up approach including more intensive tailored interventions. Between face-to-face visits, we planned to support patients with several BCTs delivered by the SMILeApp, e.g., *self-monitoring of the behavior* (Table 8).

The intervention draft was discussed with alloSCT patients and caregivers to prioritize functionalities. This process will later guide the order of the software and intervention development. Patients agreed unanimously that the SMILeApp should ideally include a current and complete medication plan that could be automatically updated after every change to the medication regimen. Patients considered this as a feasible way to confirm their medication intake after every intake. Most participants also considered it acceptable and helpful to receive a reminder for data entry once a day at a user-defined time, with graphical feedback for entered values.

All findings arising from steps 4 to 8 are presented in Table 8 which also indicates which COM-B and TDF domains are influenced by each intervention function and policy category, as well as the resulting BCTs and modes of delivery.

### Additional stage 4: SMILe care model prototype development

The translation of the SMILe intervention into an eHealth solution will enable delivery of important BCTs, e.g., self-monitoring and feedback on target behaviors to patients anywhere (e.g., in their homes or workplaces) at any time [2, 3]. Therefore, after working through the three stages and eight core steps of the BCW, we developed a fourth stage – SMILe Care Model Prototype Development – which includes two steps, both of which are based on agile software development techniques (Fig. 2). Developed to facilitate early integration within the target setting, a highly iterative methodology and the inclusion of end-user feedback in the development process ensures the software’s usefulness to patients, caregivers and clinicians alike [35].

#### Additional step 9: prepare digitalization

To prepare for digitization, step 9 deals with the formulation of user stories [35]. User stories are a means to capture requirements that should be delivered by a software product. This makes their stories a valuable basis for discussion between software developers and intervention developers [35]. User stories are an opportunity for researchers to provide key information to software developers about important functionalities that are needed in the app. They will not directly appear in the app, only by means of the functionality implemented. The goal of user stories it to provide key information between researchers and software developers and, thus, are not communicated to patients.

While user stories can be structured in numerous ways, the role-feature-reason format is most popular [35]. This begins with a structured sentence (As a … (person, e.g., *allo-SCT patient*), I want … (action, e.g., *keep track of my medication intake*), so that … (expected outcome, e.g., *I know if I take my medication correctly*)) describing a possible software function. Structuring the essential elements in a standard form eases the translation of the corresponding BCTs into the SMILeApp [35]. The concrete realization of a user story is decided in close consultation with the software development team.

**Results.** To ensure the translation of the most effective BCTs into our eHealth component, we wrote 17 user stories to the software developers in the role-feature-reason format based on the previous findings (Table 9). Following the principles of agile software development, continuous end-user testing and user-centered design, the needs and priorities these stories suggest will help the designers first to develop mock-ups of new modules for early user testing, then to ensure that later versions of the app meet user needs as fully as possible [35]. A separate paper will describe the development of the technology aspects in detail (in preparation).

**Table 9:** User stories according to the BCT

BCT	Description	User stories
1.1. Goal setting (behavior)	Goals will be set together with patient to take the medication correctly with a deviation < 2 h.	As a patient I want to be reminded of my set goals (which were set during visit) on a self-determined interval (e.g. daily / once a week) so that I am aware of my goal and know what to target at.
1.4. Actionplanning	Patients will be encouraged to prepare a plan how to deal with barriers (e.g. have a travel set of their medication prepared and to take it with them when leaving home).	As a patient I want to have reliable information how to plan my expected actions (e.g. leaving home, travelling, eating outside) so that I do not forget the necessary preparations.
2.3. Self-monitoring of behavior	Instructions on checking the medication plan daily and confirming medication intake daily. Instructions on how to control this monitoring on their own.	As a patient I want to self-monitor whether I take my medication as prescribed so that I know whether I take the drugs correctly.
2.2. Feedback on behavior	Give patients feedback about on how many days they correctly managed their medication intake. Give patients feedback about on how many days their time of medication intake was correct.	As a patient I want to get feedback whether I take my medication sufficiently as prescribed so that I can be sure that I take the medication correctly
4.1. Instruction on how to perform behavior	Training on how to read the medication plan and prepare their medication correctly accordingly.	As a patient I want to know how to prepare my medication so that I can do it on my own correctly
5.1. Information about health consequences	Oral and written information about effects, side-effects of medication. Oral and written information about consequences of non−/adherence.	As a patient I want to find information on what my medication is for and will happen, if I do (not) take it as prescribed (incl. wrong time) so that I know the importance of doing it correctly.
6.1. Demonstration of behavior	Demonstrate how to read medication plan and prepare the medication.	As a patient I want to get explained how to use the electronic medication plan so I can check when I have forgotten.
7.1. Electronic prompts/cues	Storage of medication in clear visible places (e.g., next to the coffee machine, TV), to prompt medication intake. Use of an electronic reminder (for medication intake, goal) on the App with a preferred signal (e.g., alarm tone, picture).	As a patient I want to get a reminder when I need to take my medication and don’t forget to take it. As a patient I want to customize my app (e.g. different tones, signals, colours, pictures) so that I connect medication intake with a positive feeling.
1.2. Problem solving	Identify together with patient what could be barriers to take the medication correctly, and discuss ways in which they could help overcome them. Learn about most common side-effects and fitting interventions to take.	As a patient I want to have the opportunity to call a qualified health care provider if there are unexpected barriers (which were not discussed face-to-face) so that I get support in challenging situations. As a patient I want to be able to signal the CC that she/he should call back when she/he has time so that I get help without disturbing the CC in an unsuitable situation.
1.6. Discrepancy between behavior & goal	Point out if the recorded number / time of medication intake does not fit to the goal set.	As a patient I want to get a signal if the recorded number / time of medication intake does not fit to the goal set so that I realize that I have to change my behavior.
7.1. Electronic prompts/cues	*Medication plan in app*	As a patient I want to have my medication plan in the app so that I can look up my medication on my smartphone. As a patient I want to be able to update the medication plan in the app when the prescription of physician changes so that I have a current medication plan on my smartphone.
9.1. Message given by followed person	Present a speech / statement / message given by followed patients or recognized transplant professional to emphasize the importance of taking the medication correctly at the correct time.	As a patient I want to learn from a qualified health care provider or a peer why the medication intake and timing is so important so that I am aware of its importance.
15.1. Written persuasion about capability	Motivation when confirming medication intake / closing the app / pop-up	As a patient I want to get a motivational feedback that I can successfully perform the behavior so that I feel capable to manage the correct medication intake.
15.3. Focus on past success	Explore with patients difficult circumstances in which patients nevertheless managed medication intake.	As a patient I want to be able to record occasions with correct medication intake in the App so that I feel confident to be successful again.

#### Additional step 10: prepare CC intervention

Based on the previous steps of the BCW, we wrote a comprehensive intervention protocol for the intervention’s face-to-face CC visits. This protocol describes every face-to-face visit in terms of *when*, *by whom* and to *whom* it should be delivered (patient ± caregiver(s)), as well as *which BCTs* should be applied in what order and which methods should be used. To ensure standardized, reproducible intervention delivery, descriptions and terminology adhered closely to the BCT taxonomy. The intervention protocol was written in alignment with implementation science lens, meaning, that the intervention has been designed using the previously described implementation science methods (e.g. contextual analysis, stakeholder involvement) in such a way that it can be implemented and used in real world settings in the future.

**Results.** The complete protocol consisted of 70 pages for all modules with the detailed description of the content to be used for education, training and supervision of the CC. We also wrote a short version with 49 pages for all modules to be used by the CC during the face-to-face visits as a checklist.

We initially planned to implement and test the whole medication adherence module via both, the CC (face-to-face visits) and the SMILeApp in an RCT. However, due to lack of time resources, the current version of the SMILeApp does not include medication adherence support yet. For this reason, in the current RCT, which started at the beginning of 2020 at the University Hospital of Freiburg im Breisgau (FiB), only the face-to-face visits of the medication adherence module are implemented and tested. This downsized version of the care model is called the SMILe-V1 Care Model Prototype–FiB as it is tested at the University Hospital of FiB. Our prepared user stories will be prioritized and translated into the next version of the SMILeApp to be implemented and evaluated in combination with the CC’s face-to-face visits in an RCT at the University Hospital of Basel (USB). This full version of the care model will be called the SMILe-V2 Care Model Prototype–USB.

## Discussion

To our knowledge, this paper is the first to provide an example of how to develop a theory-based medication adherence intervention for translation into an eHealth system. Our work, embedded in an implementation science approach, applied the principles recommended by the BCW, and particularly of the COM-B model at its center. This framework’s major strengths are its multilevel perspective and the explicit inclusion of emotional factors, which tend to be less prominent or absent in other behavioral theories [34]. While other authors have used the BCW to develop eHealth facilitated behavior change interventions, none have described their development process (including the translation of an intervention into an eHealth application) in this detail, and many have not included patients or incorporated them only at a late stage of intervention development [37, 38, 67].

Worldwide, health care services and providers are moving in the direction of digitization [68]. The number of newly released health related apps is currently growing by over 200 per day: between 2015 and 2018, the number available in the top app stores almost doubled to nearly 320′000 [24]. Many aim to support patients in medication adherence [22] and some have showed promising results in RCTs in various populations [14, 16, 17]. In the development of an eHealth facilitated intervention, it is crucial to rely on the theory, consider the most recent evidence, integrate information on the context where the intervention or app will be applied and involve all major stakeholders – especially patients – from the earliest stages [24, 28, 69, 70]. And while theory-based interventions are most likely to improve medication adherence [28, 69], extremely few apps are developed following these recommendations.

To exacerbate the problem, producers typically supply limited or no information on the processes either of their apps’ implementation or of their results in real-life settings [16, 29]. This paper addresses these shortcomings using the example of the SMILe integrated care model’s medication adherence intervention module. Unlike many existing eHealth tools, our intervention development incorporated extensive end-user involvement (i.e., of patients, caregivers, clinicians), enhancing its relevance regarding implementation in real-life settings. Our goal was not simple to develop the best possible intervention to provide the basis for digitalization and medication adherence interventional components, but also to maximize the likelihood of its successful implementation and sustainability in daily clinical alloSCT follow-up care.

This paper also presents a first step towards bridging the gap between trial and real-world contexts in the development and implementation of a medication adherence module: it describes how to combine empirical evidence with contextual data [36] as the foundation of an eHealth facilitated intervention. The involvement of all relevant stakeholders was indispensible for this module’s durable implementation into clinical practice [27, 34].

In addition, by developing user stories based on end-user needs and feedback, we combined the central principles of Implementation Science – sensitivity to context, building on an existing evidence base, extensive stakeholder involvement – with the principles of agile software development and user-centered design [35, 70]. With the result – an iterative, incremental, user-centered approach with early connection to the target context – we intend to speed the translation of cutting-edge research findings into routine use not only by clinicians but also by the target (alloSCT patient) population [24, 70]. By involving the various stakeholder groups at each appropriate stage, we ensured that the intervention would fit the needs of end-users and be feasible for sustainable use in clinical practice. This fact alone sets our eHealth tools apart from others, the vast majority of which are typically developed by software designers with little or no input from health care research teams [24].

The BCW is a relatively novel multilevel behavioral framework that both explains and provides a stable framework for intervention development. Building for our work upon the evaluation of correlates/determinants of medication adherence intervention development, not only does it include cognitive patient-level factors, it also explicitly includes emotional factors that are not prominent in currently prevalent health behavior models, e.g., the Integrated Model of Behavioral prediction [34, 71].

By assisting in the selection of appropriate behavior change interventions [34], the BCW provides invaluable guidance for intervention development. And by drawing from diverse information sources it allows the combination of quantitative and qualitative evidence, the results of contextual analysis and stakeholder involvement [34], and the researchers’ clinical expertise. The full range of these sources informed our intervention development.

Integral to the BCW, the BCT taxonomy [72] provides standardized language to label even the smallest units of behavioral change interventions. In addition to enhancing the reporting and communication of complex interventions’ content and enhancing their replicability, this level of standardization facilitates meta-analysis, especially as it is used to detect the best-performing intervention components.

Although the BCW is arguably an excellent foundation for intervention development, we added one stage to it at the end of our development process. To speed and simplify translation of the medication adherence module into an eHealth application, we recommend and describe the formulation of user stories as a bridge between intervention developers and software developers. Congruent with the BCW’s aims, agile software development focuses both on stakeholder involvement and on the adaption of the software according to the needs of its end-users [35].

One notable challenge regarding the use of the BCW is that, even while its developers provide a step-by-step process for intervention development, they tend to provide only brief descriptions of the links between those steps, which can make following their recommendations rather challenging especially for those who use the framework for the first time. Regarding applying COM-B to our specific research question, we faced the issue that there is very few evidence regarding medication adherence in alloSCT. Therefore, we had to expand our searches on similar populations. Additionally, following the steps left us with more potential options (i.e., BCTs) than could possibly be realized from a resource and logistical perspective. Future refinement of the BCW could help to overcome this challenge by providing more guidance on which of the possible BCTs would be most appropriate. In our example we based the selection and reduction to the most important and promising options on the feedback of the stakeholders to promote feasible implementation: We applied the APEASE criteria to each possible BCT and discussed which possible app functionalities would specifically help stem cell transplant patients and their caregivers with decision-making and prioritization. This approach ensured that the intervention would fit the needs and preferences of the end-users.

Application of theory is facilitated by examples. While our example focuses directly on the development of behavioral intervention and eHealth app, it and its underlying theory can also be applied to the adaptation of existing interventions and apps to new contexts. Nevertheless, a strong knowledge of the theory is crucial and needs to be firmly in place before starting the development process. To bring all the needed competencies and perspectives to the table, an interdisciplinary team and stakeholder involvement are essential.

Our international multidisciplinary research team consists of 31 researchers and clinicians, two of whom were members of the BCW development team for the medication adherence module. Several of our researchers also went to London to follow summer courses offered by the group led by Susan Michie, who developed the BCW. In all, development of the SMILe intervention modules required an investment of more than 1 year. Therefore, we advise other researchers that, to prepare adequately for this task, they should combine readings of papers and books with formal training. Another valuable option would be to collaborate with more experienced researchers to peer support this process.

It is still unknown, whether a theory-based developed intervention module such as ours is able to improve medication adherence in alloSCT. In addition, it is unclear whether an analogue or digital intervention, or even the combination of both is the most successful which needs further investigation. Our study’s results will support agile technology development of the SMILe medication adherence module as part of the SMILe integrated care model. The SMILe-V1 Care Model Prototype–FiB (without medication adherence in the SMILeApp) and SMILe-V2 Care Model Prototype–USB (with medication adherence in the SMILeApp) will subsequently be implemented and tested as part of an implementation science study to address these knowledge gaps (https://smile.nursing.unibas.ch/). Once the SMILe Care Model Prototype has proven its effectiveness, it can be sustainably implemented in clinical practice as it was developed under consideration of implementation science aspects (e.g. feasibility and acceptability).

### Limitations

Our development of the eHealth component of our care model was subject to certain restrictions. For example, as contextual factors such as data security legislation are very strict in Switzerland and the EU, certain proposed app functions could not be included. Even if qualitative literature and the involved patients had certain priorities regarding the functionality of the SMILeApp, other factors such as the legal framework could force different sequences of the app development process. However, the whole medication adherence module is more than just the eHealth part, why a first testing of the intervention is still possible.

## Conclusion

As intended, this paper describes the theory-driven development, based on the BCW, of a medication adherence intervention module as part of the SMILe integrated care model for alloSCT patients, which includes a patient-centered app and introduces one new care team role: a CC. The associated study’s results are currently in use for agile technology development, employing a user-centered design approach, of the innovative SMILe integrated care model’s medication adherence module. The overall care model will be implemented and tested as part of a planned implementation science study. While the methods described are applied to this particular medication adherence intervention, as they follow BCW recommendations, they can be adapted to the development of a wide range of behavior-targeted eHealth applications.

## Supplementary information

**Additional file 1.**

## Data Availability

The data of the previously performed contextual analysis included in the development of the intervention were recently published by LL and our research group [36]. As the original data is personal and cannot be completely anonymized, it cannot be made available for open access. Other data analyzed and synthesized are available from the corresponding author upon request.

## Abbreviations

AlloSCT: Allogeneic stem cell transplantation
BCT: Behavior Change Techniques
BCW: Behavior Change Wheel
CC: Care-coordinator
COM-B: Capability-Opportunity-Motivation-Behavior Model
CReDECI 2: Criteria for Reporting the Development and Evaluation of Complex Interventions in healthcare: revised guideline
EM: Electronic monitoring
EMERGE: ESPACOMP Medication Adherence Reporting Guideline
ESPACOMP: European Society for Patient Adherence, Compliance, and Persistence
FiB: Freiburg im Breisgau
GvHD: Graft-versus-Host Disease
MNA: Medication non-adherence
RCT: Randomized controlled trial
TDF: Theoretical Domains Framework
TIDieR: Template for Intervention Description and Replication
USB: University Hospital of Basel

## Footnotes

1 Although implementation adherence uses the same word, this does not equate with implementation in the context of implementation science.
