# Supplementary material for: Theory-driven development of a medication adherence intervention delivered by eHealth and transplant team in allogeneic stem cell transplantation: the SMILe implementation science project
Source: BMC Health Serv Res. 2020 Sep 2;20:827. doi: 10.1186/s12913-020-05636-1 (PMC7465386; doi:10.1186/s12913-020-05636-1)
Supplement: Supplementary file 1 — Additional file 1. [file 12913_2020_5636_MOESM1_ESM.docx]

# Appendix

### Search diagram 1

Scoping review for quantitative literature on the definitions, prevalences and consequences of medication non-adherence in alloSCT:

- Databases: PubMed, Cochrane, CINAHL, Embase
- Search terms (all fields): *medication adherence* AND *bone marrow transplantation* AND *prevalence* AND *treatment outcome* and synonyms in different combinations
- Date of literature search: 02.06.2018
- Search ended: May 2018
- Method: After removal of duplicates, the identified titles and abstracts were screened by the first author. From the selected studies, data providing evidence on definition, prevalence and consequences of medication non-adherence were extracted and categorized by the first author according to three questions: *What is the problem / behavior?*; *Where does it occur?;* and *Who is / Is our target group involved?*

Articles identified through database searching (n = 426)

PubMed (n = 140)

Cochrane (n = 11)

CINAHL (n = 87)

Embase (n = 188)

Articles after duplicates (n = 28) removed

(n = 398)

Screened for relevance to topic

(n = 398)

Excluded (n = 386)

82 = paediatric studies

82 = other oncological conditions

79 = medical disease management

78 = other (chronic) conditions

42 = other behaviors

5 = research collaboration reports

5 = ethical research questions

4 = trials with animal

4 = quality management

3 = meeting abstracts/reports

2 = study protocols

Full-text articles assessed for eligibility

(n = 12)

Articles included in qualitative synthesis

(n = 12)

**Included studies:**

Bixby, D. L. (2013). Managing inadequate responses to frontline treatment of chronic myeloid leukemia: a case-based review. *Cancer treatment reviews*, *39*(3), 241-251.

De Geest, S., Abraham, I., Moons, P., Vandeputte, M., Van, J. C., Evers, G., ... & Vanhaecke, J. (1998). Late acute rejection and subclinical noncompliance with cyclosporine therapy in heart transplant recipients. *The Journal of heart and lung transplantation: the official publication of the International Society for Heart Transplantation*, *17*(9), 854-863.

Gresch, B., Kirsch, M., Fierz, K., Halter, J. P., Nair, G., Denhaerynck, K., & De Geest, S. (2017). Medication nonadherence to immunosuppressants after adult allogeneic haematopoietic stem cell transplantation: a multicentre cross-sectional study. *Bone marrow transplantation*, *52*(2), 304-306.

Hoodin, F. (1995). Psychological and behavioral correlates of medical adherence among adult bone marrow transplant recipients.

Kirsch, M., Götz, A., Halter, J. P., Schanz, U., Stussi, G., Dobbels, F., & De Geest, S. (2014). Differences in health behaviour between recipients of allogeneic haematopoietic SCT and the general population: a matched control study. *Bone marrow transplantation*, *49*(9), 1223-1230.

Lehrer, J., Brissot, E., Ruggeri, A., Dulery, R., Vekhoff, A., Battipaglia, G., ... & Antignac, M. (2018). Medication adherence among allogeneic hematopoietic stem cell transplant recipients: a pilot single-center study. *Bone marrow transplantation*, *53*(2), 231-233.

Montesinos, P., Rodríguez-Veiga, R., Boluda, B., Martínez-Cuadrón, D., Cano, I., Lancharro, A., ... & Lorenzo, I. (2015). Incidence and risk factors of post-engraftment invasive fungal disease in adult allogeneic hematopoietic stem cell transplant recipients receiving oral azoles prophylaxis. *Bone marrow transplantation*, *50*(11), 1465-1472.

Morrison, C. F., Martsolf, D. M., Wehrkamp, N., Tehan, R., & Pai, A. L. (2017). Medication adherence in hematopoietic stem cell transplantation: a review of the literature. *Biology of Blood and Marrow Transplantation*, *23*(4), 562-568.

Pinsky, B. W., Takemoto, S. K., Lentine, K. L., Burroughs, T. E., Schnitzler, M. A., & Salvalaggio, P. R. (2009). Transplant outcomes and economic costs associated with patient noncompliance to immunosuppression. *American Journal of Transplantation*, *9*(11), 2597-2606.

Talati, C., Ontiveros, E. P., Griffiths, E. A., Wang, E. S., & Wetzler, M. (2015). How we will treat chronic myeloid leukemia in 2016. *Blood reviews*, *29*(2), 137-142.

Vrijens, B., De Geest, S., Hughes, D. A., Przemyslaw, K., Demonceau, J., Ruppar, T., ... & Matyjaszczyk, M. (2012). A new taxonomy for describing and defining adherence to medications. *British journal of clinical pharmacology*, *73*(5), 691-705.

Welch, M., & Kaled, E. (2013). Ensuring optimal adherence to BCR-ABL1 tyrosine kinase inhibitor therapy for chronic myeloid leukemia. *Community Oncol*, *10*(5), 138-46.

### Search diagram 2

Scoping review for qualitative literature on barriers and facilitators to medication adherence in alloSCT and transplant patients:

- Database: PubMed
- Search string (all fields): *adherence* AND *transplant* AND *qualitative*
- Date of literature search: 09.06.2018
- Search until: May 2018
- Method: After duplicates were removed, the identified titles and abstracts were screened blinded and full texts further analyzed by the first and last author. In case of different appraisal, the selection was discussed until consensus was reached. The same procedure was used for the next step
- From the selected studies, data providing evidence on the experience of barriers and facilitators to medication adherence were extracted and categorized according to the COM-B.

Articles identified through database searching in PubMed

(n = 76)

Articles after duplicates (n = 0) removed

(n = 76)

Screened for relevance to topic

(n = 76)

Excluded (n = 60)

20 = paediatric /adolescent patients

16 = not medication adherence

14 = not transplant setting

3 = quantitative analysis

3 = study protocols

2 = studies about donors

2 = programme development/evaluation

Full-text articles assessed for eligibility

(n = 16)

Articles included in qualitative synthesis

(n = 16)

**Included studies:**

Boaz, A., & Morgan, M. (2014). Working to establish ‘normality’post-transplant: A qualitative study of kidney transplant patients. *Chronic illness*, *10*(4), 247-258.

Cooke, L., Chung, C., & Grant, M. (2011). Psychosocial care for adolescent and young adult hematopoietic cell transplant patients. *Journal of psychosocial oncology*, *29*(4), 394-414.

Israni, A., Dean, C., Kasel, B., Berndt, L., Wildebush, W., & Wang, C. J. (2016). Why do patients forget to take immunosuppression medications and miss appointments: can a mobile phone app help?. *JMIR public health and surveillance*, *2*(1), e15.

Ivarsson, B., Ekmehag, B., & Sjöberg, T. (2013). Patients’ experiences of information and support during the first six months after heart or lung transplantation. *European journal of cardiovascular nursing*, *12*(4), 400-406.

Jamieson, N. J., Hanson, C. S., Josephson, M. A., Gordon, E. J., Craig, J. C., Halleck, F., ... & Tong, A. (2016). Motivations, challenges, and attitudes to self-management in kidney transplant recipients: a systematic review of qualitative studies. *American Journal of Kidney Diseases*, *67*(3), 461-478.

Janelle, C., O’Connor, K., & Dupuis, G. (2016). Evaluating illness representations in heart transplant patients. *Journal of health psychology*, *21*(9), 1850-1859.

Low, J. K., Crawford, K., Manias, E., & Williams, A. (2017). Stressors and coping resources of Australian kidney transplant recipients related to medication taking: a qualitative study. *Journal of clinical nursing*, *26*(11-12), 1495-1507.

Low, J. K., Crawford, K., Manias, E., & Williams, A. (2016). A compilation of consumers’ stories: the development of a video to enhance medication adherence in newly transplanted kidney recipients. *Journal of advanced nursing*, *72*(4), 813-824.

Muduma, G., Shupo, F. C., Dam, S., Hawken, N. A., Aballéa, S., Odeyemi, I., & Toumi, M. (2016). Patient survey to identify reasons for non-adherence and elicitation of quality of life concepts associated with immunosuppressant therapy in kidney transplant recipients. *Patient preference and adherence*, *10*, 27.

O'Grady, J. G., Asderakis, A., Bradley, R., Burnapp, L., McPake, D. M., Perrin, M., ... & Wilson, L. C. (2010). Multidisciplinary insights into optimizing adherence after solid organ transplantation. *Transplantation*, *89*(5), 627-632.

Pinter, J., Hanson, C. S., Craig, J. C., Chapman, J. R., Budde, K., Halleck, F., & Tong, A. (2016). ‘I feel stronger and younger all the time’—perspectives of elderly kidney transplant recipients: thematic synthesis of qualitative research. *Nephrology Dialysis Transplantation*, *31*(9), 1531-1540.

Rebafka, A. (2016). Medication Adherence After Renal Transplantation—a Review of the Literature. *Journal of renal care*, *42*(4), 239-256.

Tong, A., Howell, M., Wong, G., Webster, A. C., Howard, K., & Craig, J. C. (2011). The perspectives of kidney transplant recipients on medicine taking: a systematic review of qualitative studies. *Nephrology Dialysis Transplantation*, *26*(1), 344-354.

Tong, A., Morton, R. L., & Webster, A. C. (2016). How qualitative research informs clinical and policy decision making in transplantation: A review. *Transplantation*, *100*(9), 1997-2005.

Williams, A., Crawford, K., Manias, E., Ellis, C., Mullins, K., Howe, K., ... & Van Hardeveld, E. (2015). Examining the preparation and ongoing support of adults to take their medications as prescribed in kidney transplantation. *Journal of evaluation in clinical practice*, *21*(2), 180-186.

Williams, A., Low, J. K., Manias, E., & Crawford, K. (2016). The transplant team's support of kidney transplant recipients to take their prescribed medications: a collective responsibility. *Journal of clinical nursing*, *25*(15-16), 2251-2261.

### Search diagram 3

Systematic search for quantitative literature about interventions to support medication adherence in alloSCT patients:

- Data bases: PubMed, Cochrane, CINAHL, Embase
- Search terms (all fields): (*stem cell transplantation* OR *hematopoietic stem cell transplantation* OR *bone marrow transplantation* OR *allogeneic hematopoietic stem cell transplantation* OR *HSCT* OR *HSCT survivors*) AND (*nursing process* OR *patient education as topic* OR *education* OR *teaching* OR *telemedicine* OR *drug monitoring* OR *reminder systems* OR *nursing intervention* OR *eHealth* OR *chronic care model* OR *behavior change wheel*) AND (*medication adherence* OR *patient compliance* OR *medication systems* OR *medication therapy management* OR *BAASIS* OR *medication implementation* OR *medication persistence*)
- Date of literature search: 16.06.2018
- Search ended: May 2018
- Method: After duplicates were removed, the identified titles and abstracts were screened by the first author. From the selected studies, data providing evidence on interventions to support medication adherence were extracted by the first author and reported in evidence tables in nine sections: *author/year, aims, design/setting/sample/sampling, measurement/variable, results, limitations, population characteristics, intervention description, and theoretical framework*.

Articles identified through database searching (n = 439)

PubMed (n = 89)

Cochrane (n = 95)

CINAHL (n = 28)

Embase (n = 227)

Articles after duplicates (n = 30) removed

(n = 409)

Screened for relevance to topic

(n = 409)

Excluded (n = 397)

84 = pharmacological studies

71 = other outcomes

56 = paediatric studies

43 = other chronic conditions

39 = other oncological conditions

37 = medical disease management

16 = meeting abstracts

11 = Economic studies

9 = about central venous catheters

9 = ethics studies

7 = trials with animal

6 = Full Text not available

4 = accreditation evaluations

2 = adherence to vaccination

2 = in systematic review included

1 = editorial

Full-text articles assessed for eligibility

(n = 12)

Full-text articles excluded (n = 1)

1 = intervention not described

Articles included in qualitative synthesis

(n = 11)

**Included studies:**

Bixby, D. L. (2012). Managing inadequate responses to frontline treatment of chronic myeloid leukemia: A case-based review. *Cancer Treatment Reviews*, 39(3), 241-251. doi: 10.1016/j.ctrv.2012.04.010

Cooke, L., Chung, C., & Grant, M. (2011). Psychosocial care for adolescent and young adult hematopoietic cell transplant patients. *Journal of Psychosocial Oncology*, 29(4), 394-414.

De Bleser, L., Matteson, M., Dobbels, F., Russell, C., & De Geest, S. (2009). Interventions to improve medication‐adherence after transplantation: a systematic review. *Transplant International*, 22(8), 780-797. doi: 10.1111/j.1432-2277.2009.00881.x

Demonceau, J., Ruppar, T., Kristanto, P., Hughes, D. A., Fargher, E., Kardas, P., ... Vrijens, B. (2013). Identification and assessment of adherence-enhancing interventions in studies assessing medication adherence through electronically compiled drug dosing histories: a systematic literature review and meta-analysis. *Drugs*, 73(6), 545-562. doi: 10.1007/s40265-013-0041-3

Dobbels, F., De Bleser, L., Berben, L., Kristanto, P., Dupont, L., Nevens, F., ... & De Geest, S. (2017). Efficacy of a medication adherence enhancing intervention in transplantation: The MAESTRO-Tx trial. *The Journal of Heart and Lung Transplantation*, *36*(5), 499-508. doi: 10.1016/j.healun.2017.01.007

Kirsch, M., Berben, L., Johansson, E., Calza, S., Eeltink, C., Stringer, J., ... & De Geest, S. (2014). Nurses' practice patterns in relation to adherence‐enhancing interventions in stem cell transplant care: a survey from the Nurses Group of the European Group for Blood and Marrow Transplantation. *European Journal of Cancer Care*, 23(5), 607-615. doi: 10.1111/ecc.12172

Low, J. K., Williams, A., Manias, E., & Crawford, K. (2014). Interventions to improve medication adherence in adult kidney transplant recipients: a systematic review. *Nephrology Dialysis Transplantation*, 30(5), 752-761. doi: 10.1093/ndt/gfu204

Schmid, A., Hils, S., Kramer‐Zucker, A., Bogatyreva, L., Hauschke, D., De Geest, S., & Pisarski, P. (2017). Telemedically Supported Case Management of Living‐Donor Renal Transplant Recipients to Optimize Routine Evidence‐Based Aftercare: A Single‐Center Randomized Controlled Trial. *American Journal of Transplantation*, 17(6), 1594-1605. doi: 10.1111/ajt.14138

Talati, C., Ontiveros, E. P., Griffiths, E. A., Wang, E. S., & Wetzler, M. (2015). How we will treat chronic myeloid leukemia in 2016. *Blood reviews*, 29(2), 137-142. doi:10.1016/j.blre.2014.12.003

Welch, M. A., & Kaled, E. S. (2013). Ensuring optimal adherence to BCR-ABL1 tyrosine kinase inhibitor therapy for chronic myeloid leukemia. *Community Oncology*, 10(5), 138-146. doi: 10.12788/j.cmonc.0029

Wesley, K. M., & Fizur, P. J. (2015). A review of mobile applications to help adolescent and young adult cancer patients. *Adolescent Health, Medicine and Therapeutics*, 6, 141-148. doi: 10.2147/AHMT.S69209
